# The Evaluation of Different Radiological Measurement Parameters of the Degree of Collapse of the Vertebral Body in Vertebral Compression Fractures

**DOI:** 10.1155/2019/4021640

**Published:** 2019-05-08

**Authors:** Wei-En Hsu, Kuo-Chih Su, Kun-Hui Chen, Chien-Chou Pan, Wen-Hsien Lu, Cheng-Hung Lee

**Affiliations:** ^1^Department of Orthopedics, Taichung Veterans General Hospital, Taichung, Taiwan; ^2^Department of Medical Research, Taichung Veterans General Hospital, Taichung, Taiwan; ^3^Department of Biomedical Engineering, Hung Kuang University, Taichung, Taiwan; ^4^Department of Rehabilitation Science, Jenteh Junior College of Medicine, Nursing, and Management, Miaoli County, Taiwan; ^5^Department of Orthopedics, Feng Yuan Hospital, Taichung, Taiwan; ^6^Department of Food Science and Technology, Hung Kuang University, Taichung, Taiwan

## Abstract

For compression fracture, vertebral body height loss (VBHL) and kyphotic angle (KA) are two important imaging parameters for determining the prognosis and appropriate treatment. This study used previous measurement methods to assess the degree of VBHL and KA, compare and examine differences between various measurement methods, and examine the correlation between relevant measurement parameters and intravertebral cleft (IVC) in the vertebral body. The radiographic images (lateral view of the T-L spine) of 18 patients with a single-level vertebral compression fracture were reviewed. We measured 9 characteristic lengths and angles on plain radiographs, including anterior vertebral height (AVH) and AVH of the adjacent upper and lower levels, middle vertebral height (MVH) and MVH of the adjacent upper and lower levels, posterior vertebral height (PVH), and vertebral body width, and assessed 6 parameters, including vertebral compression ratio (VBCR), percentage of anterior height compression (PAHC), percentage of middle height compression (PMHC), kyphotic angle (KA), calculated kyphotic angle (CKA), and IVC. The results showed that VBCR is a simple and rapid method of VBHL assessment, but it may result in an underestimation of the degree of VBHL compared to PAHC. When PMHC < 40% or kyphotic angle > 15°, the probability of IVC occurring on the vertebral body was higher which means the higher risk of vertebral body instability. The results of this study could provide a reference for surgeons when using imaging modalities to assess the degree of vertebral body collapse.

## 1. Introduction

As the population ages, the prevalence of osteoporosis also gradually increases, and the resulting medical cost increases every year. Vertebral compression fracture is one of the important complications of osteoporosis [1, 2]. In clinical practice, acute lower back pain is a classical presentation of the disease. Among imaging modalities, radiography is the most widely used tool for the diagnosis and assessment of compression fractures. Vertebral body height loss (VBHL) and kyphotic angle (KA) are two important imaging parameters for determining the prognosis and appropriate treatment [3]. The degrees of collapse of the vertebral body may affect the direction of treatment (nonsurgical management, like rest, bracing, and pain control drug, or surgery, like vertebra augmentation, instrumentation, or fusion). As VBHL increases, the vertebra may become more unstable. In addition to its effects on stability, KA can negatively affect sagittal alignment. Data have shown that a KA of >15°–30° or height loss of >50% resulted in vertebral body instability, which may require a more invasive treatment such as vertebral augmentation [4–6].

The definition and measurement methods for these two important imaging parameters are still controversial, and no conclusion has been reached thus far [7]. Previously, researchers used different measurement methods to assess the degree of vertebral body collapse by using posterior wall height as the reference vertebral body compression ratio (VBCR) or the percentage of anterior height compression (PAHC), which uses the mean height of segments adjacent to a healthy vertebral body as the reference value [8–11]. However, differences in the range of vertebral body collapse may result in measurement errors. In addition to the degree of vertebral body collapse, the intravertebral cleft (IVC) can represent osteonecrosis of the vertebral body, which has a higher probability of progressing to advanced kyphosis [12]. However, thus far, to the best of our knowledge, no study has examined the correlation between IVC and VBHL.

The aforementioned literature demonstrates that different data will be obtained if different measurement methods are used. In addition, differences in deformity may also result in measurement errors. However, no related study has examined and analyzed the differences and limitations of different measurement methods. Therefore, the aim of this study was to use previous measurement methods to assess the degree of vertebral body collapse, compare and examine differences between various measurement methods, and examine the correlation between relevant measurement parameters and IVC in the vertebral body.

## 2. Materials and Methods

### 2.1. Collection of Materials

This study quantitatively analyzed radiographic images (lateral view of the T-L spine) obtained before single-level vertebroplasty in patients who had osteoporotic vertebral compression fractures treated between June 2013 and January 2015 at the Department of Orthopedics, Taichung Veterans General Hospital, Taiwan. The radiographic images of 18 patients who underwent single-level vertebroplasty were examined. Seven patients were male, and 11 were female. These 18 sets of radiographic images were obtained from 101 images by excluding images with poor preoperative radiographic quality, having difficulty for taking measurements, showing a compression fracture in two or more vertebral levels, showing a nonosteoporotic vertebral compression fracture (bone tumor, metastasis, or infection), or showing a previous spine surgery or combination with other surgeries (instrumentation or fusion). Clinical parameters such as age, sex, height, body weight, and vertebral level of the 18 patients were recorded.

### 2.2. Measurement of Imaging Parameters

**Figure 1:**
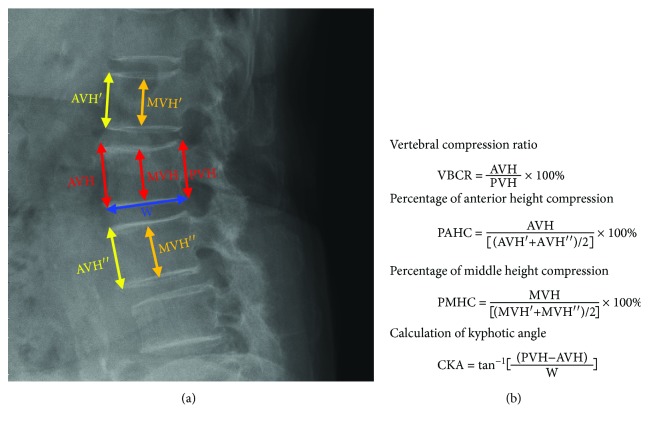
(a) This study mainly measures the characteristic lengths on plain radiographs (AVH: anterior vertebral height; MVH: middle vertebral height; PVH: posterior vertebral height; W: vertebral body width). (b) Parameters examined in this study (VBCR, PAHC, PMHC, and CKA).

**Figure 2:**
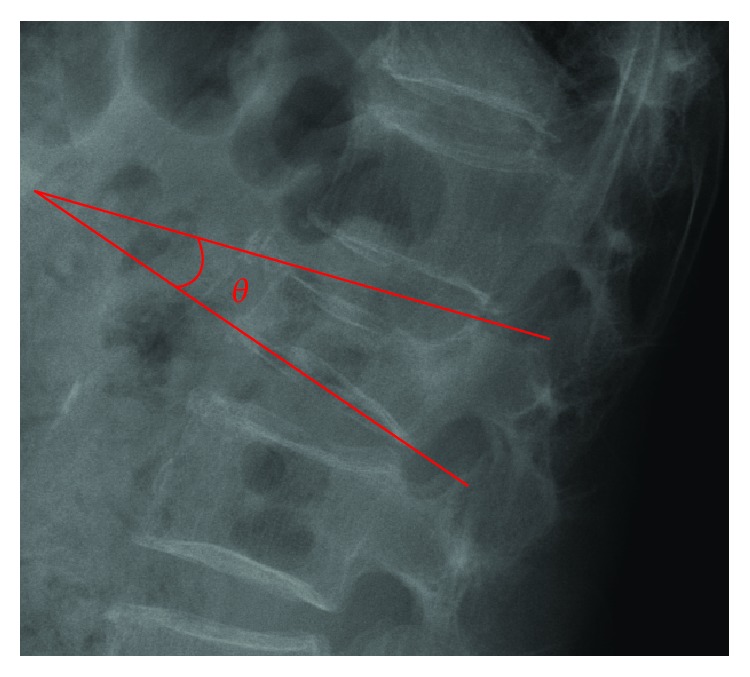
Kyphotic angle measurements.

**Figure 3:**
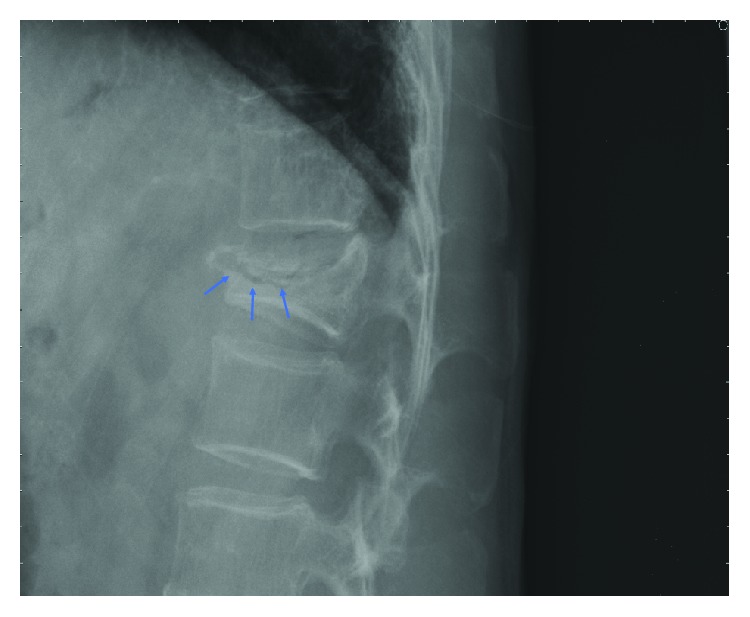
Lateral plain radiograph of the thoracolumbar spine. The arrows point to the crescent-shaped shadow in the vertebral body, that is, the IVC.

This study mainly measured 9 characteristic lengths and angles on plain radiographs. The image was digitalized and could be reviewed via professional software, SmartIris (SmartIris 1.3.0.14, Taiwan Electronic Data Processing Corp.), which contains a lot of tools, including measurement of distance and angle and zoom-in or zoom-out. With this assistance of this professional software, the image can be enlarged to show the detail which we want to measure. The distance and angle could be measured accurately. These parameters included anterior vertebral height (AVH) and AVH of the adjacent upper and lower levels (AVH′ and AVH^″^, respectively), middle vertebral height (MVH) and MVH of the adjacent upper and lower levels (MVH′ and MVH^″^, respectively), posterior vertebral height (PVH), vertebral body width (W; Figure 1), and KA (Figure 2). The lengths and angles measured using these 9 measurements were used to assess the degree of vertebral body collapse. Six observation markers were used: (1) VBCR = AVH/PVH × 100%; VBCR is mainly used to calculate the AVH-to-PVH ratio; (2) PAHC = AVH/[(AVH′ + AVH^″^)/2] × 100%; PAHC mainly calculates the ratio of AVH to the mean of AVH of the adjacent upper and lower levels; (3) percentage of middle height compression (PMHC) = MVH/[(MVH′ + MVH^″^)/2]; PMHC mainly calculates the ratio of MVH to the mean MVH of the adjacent upper and lower segments; (4) KA; (5) calculated KA (CKA) = tan^−1^[(PVH − AVH)/W]; and (6) IVC (Figure 3).

This study measured the length and angle data of the vertebral body after collapse in the 18 sets of radiographs and obtained 6 markers for examining vertebral body collapse. The analyzed markers can be used to compare the differences between the PAHC and VBCR measurements. In addition, PMHC, KA, and CKA were used to assess associations with IVC occurrence.

## 3. Results

In this study, we used 18 sets of images for radiographic (lateral view of the T-L spine) measurements.  Table 1 mainly shows the relevant data of the patients (age, sex, height, body weight, and number of images of the vertebral bodies at different levels).

**Figure 4:**
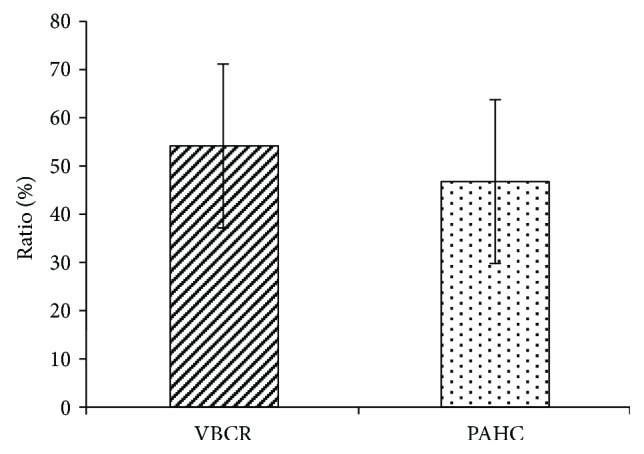
The values of VBCRs and PAHC measured from 18 sets of radiographs.

Figure 4 mainly shows the mean VBCR (range, 22.94%–84.75%; mean, 54.21%±17.13%) and PAHC (range, 20.00%–77.82%; mean, 54.21%±17.13%) of the 18 sets of radiographs. The marker results show that the VBCRs are greater than the PAHC, with differences ranging from −2.5% to 27.74%.

Table 2 shows the calculated PMHC, KA, and CKA of the 18 sets of radiographs. The PMHC ranged from 14.23% to 74.37% (mean, 46.12%±21.35%). The KAs ranged from 4.81° to 26.91° (mean, 14.95 ± 5.45). CKAs ranged from 5.00° to 23.80° (mean, 13.86 ± 6.93). In addition, in Table 2, whether IVC occurred in each group is indicated.

## 4. Discussion

In this study, we mainly used the geometric data of radiographic measurements to examine different assessment markers and assess vertebral body collapse. Of the markers used, VBCR, PAHC, and KA were based on assessment methods in previous studies, while PMHC and CKA are new parameters examined in this study.

Previous studies pointed out that most physicians usually use VBCR to assess VBHL [8]. However, the Spine Trauma Study Group recommends the use of PAHC measurement in the assessment of VBHL [9]. In this study, we observed and compared the results of VBCR and PAHC and found that VBCRs are generally higher than PAHC (−2.5% to 27.74%). The main reason for this is that compression fractures may involve the entire vertebral body, resulting in a decrease in the height of the entire vertebral body. Therefore, the use of PVH to assess vertebral body height may result in an underestimation of the degree of collapse. By contrast, PAHC mainly measures the mean of the heights of adjacent segments and will therefore not be affected by vertebral body collapse and not result in measurement errors.

In addition, our study calculated PMHC to represent the degree of vertebral body collapse. In most studies that assessed VBHL, AVH was mostly used to represent the degree of collapse in the entire vertebral body [8, 9]. However, clinical observations showed that vertebral body collapse at the middle region is often the region with the most severe collapse [13]. This may be because the bone at the middle region is softer. Therefore, we proposed PMHC as a marker of the degree of collapse at the middle region of the vertebral body. Our study also found that PMHC was associated with IVC. When PBMC was <40% (degree of collapse, >60%), the probability of IVC was higher (62.5%, 10% in the group with PMHC of >40%). Several studies suggested that IVC will increase the risk of vertebral body instability [14–16]. Therefore, we believed that in addition to PAHC, PMHC may be another important marker to assess vertebral compression fractures. However, more reliable studies are needed to elucidate the clinical role of PMHC.

In addition to being a measurement marker for assessing vertebral body collapse, KA also affects sagittal alignment [17, 18]. However, endplate deformation may sometimes occur during vertebral body collapse, and angles are not easy to measure, which can result in measurement errors. On the other hand, some researchers suggested that the endplates at the top and bottom of adjacent segments can be used to measure Cobb's angle [19]. However, this measurement method will be affected by disk deformity. In our study, we proposed the use of the anterior and posterior heights of the vertebral body and vertebral body width to estimate the kyphotic angle. This can be used as an alternative method when the angle cannot be measured. The CKA calculation results presented in this study show similar trends as the kyphotic angle measurement results in previous studies (difference, <2° in most case). However, larger errors will occur in CKA measurement during severe middle collapse of the vertebral body, as PVH and AVH are not parallel. Previous studies proposed that a reduction of 4 cm in vertebral body height will result in more than 15° of kyphotic deformity [20], while our study found that KA may be increased by 1° when there is a height difference of 7 mm between PVH and AVH. In addition, our study also found that the greater the kyphotic angle or CKA (when the angle is greater than about 15°), the greater the probability of IVC occurring at the vertebral body.

Our study used radiography for measurements, which may have some limitations. The height of adjacent segments must be used as a reference in PAHC measurements. Therefore, at present, it is only applicable to measurements for compression fractures at adjacent segments of healthy vertebral bodies. Higher-quality studies are required to determine whether this method can be extended to compression fractures in multiple segments. In addition, our study only focused on osteoporotic vertebral compression fractures and excluded compression fractures due to other factors or due to the presence of images with poor quality. Therefore, the number of reviewed images was low, and the most reviewed levels were mainly T12 and L1, which are also the most common sites for osteoporotic vertebral compression fractures. Therefore, the data obtained have reference values.

In this study, we used radiographic images to assess 6 parameters related to the vertebral body. VBCR only requires AVH and PVH measurements and is a simple and rapid method of VBHL assessment. However, when the collapse involves the posterior wall, the use of VBCR may result in an underestimation of the degree of VBHL. By contrast, although more parametric measurements are required for PAHC, it can assess VBHL with higher accuracy. More studies are expected to examine markers of severity of osteoporotic vertebral compression fractures, and a unified measurement standard is needed in the future so that clinicians can have reference data for assessment of the degree of collapse of the vertebral body and determining treatment directions.

## 5. Conclusion

We used radiographic images to measure the 9 characteristic lengths and angles of vertebral bodies to assess the degree of VBHL. These data were used to calculate the 6 assessment markers. The study results showed that values obtained with VBCR assessment were greater than those obtained with the PAHC. When the measured PMHC was <40%, the probability of observing IVC on the vertebral body was higher. When the measured KA or CKA was greater, the probability of IVC occurring on the vertebral body was higher. The results of this study can provide a reference for surgeons when using imaging modalities to assess the degree of vertebral body collapse.

## Figures and Tables

**Table 1:** Patient characteristics.

Age (years)	78.94 ± 7.92	
Sex	Male: 7, female: 11	
Height (cm)	156.7 ± 6.57	
Body weight (kg)	58.8 ± 12.90	
The number of images of the vertebral bodies at different levels	T9	1
	T12	6
	L1	8
	L3	2
	L5	1

**Table 2:** The PAHC, KA, CKA, and IVC data obtained in this study.

Imaging sample No.	PMHC (%)	KA (°)	CKA (°)	Whether IVC occurs
1	22.55	10	13.90	No
2	26.23	16	17.23	No
3	64.98	18	19.46	No
4	37.65	18	16.97	Yes
5	14.23	19	18.19	Yes
6	74.37	6	9.92	No
7	63.92	14	10.07	No
8	85.51	10	12.36	No
9	43.09	17	19.62	No
10	38.93	15	15.76	Yes
11	40.68	15	17.52	Yes
12	65.38	12	12.69	No
13	20.38	24	26.91	Yes
14	63.18	12	14.43	No
15	25.94	19	22.83	Yes
16	29.09	13	12.56	No
17	44.00	5	4.82	No
18	70.08	12	14.00	No

## Data Availability

The data used to support the findings of this study are available from the corresponding author upon request.
